# Supplementary material for: DeepFocus: fast focus and astigmatism correction for electron microscopy
Source: Nat Commun. 2024 Jan 31;15:948. doi: 10.1038/s41467-024-45042-3 (PMC10830472; doi:10.1038/s41467-024-45042-3)
Supplement: Supplementary file 1 — Supplementary Information [file 41467_2024_45042_MOESM1_ESM.pdf]

# Supplementary Information

## DeepFocus: Fast focus and astigmatism correction for electron microscopy

Schubert et al.

## Supplementary Texts

### Supplementary Text 1

We noticed that the ResNet-50 shows good performances within a limited defocus range (<15 µm aberration; 0.99 ± 1.1; n=34; EfficientNet: 1.07 ± 0.83), but a sharp error increase above, leading to an overall inferior performance compared to the other architectures (Table 1). Together with the poor performance on the tin on carbon test data (Supplementary Table 4), we speculate that this might be an overfitting effect due to the comparably small training data set and larger number of parameters (Stacked Conv.: 1,816,773; EfficientNet: 4,418,488; ResNet: 24,555,587).

### Supplementary Text 2

We performed additional convergence experiments on setup A where we tested 14 mild test aberrations sampled from a uniform distribution with value ranges between ±10 µm, ±1, ±1 (wd, stig x, stig y; example trajectory shown in Supp. Fig. 5a), of which all 14 trials converged. The current focus parameters were perturbed with noise drawn from a uniform distribution within (±2 µm, ±0.5, ±0.5) in case there was no improvement within the last 5 iterations (at most every 5 iterations). The focus baseline was found using the model from Fig. 2a with n=10 patch-pairs. The stopping threshold was found by scoring the auto-focused image, multiplying it by 1.1 and restricting it to ≥0.001 (all such obtained scores were found to be between 0.001 and 0.0014).

# Supplementary Figures

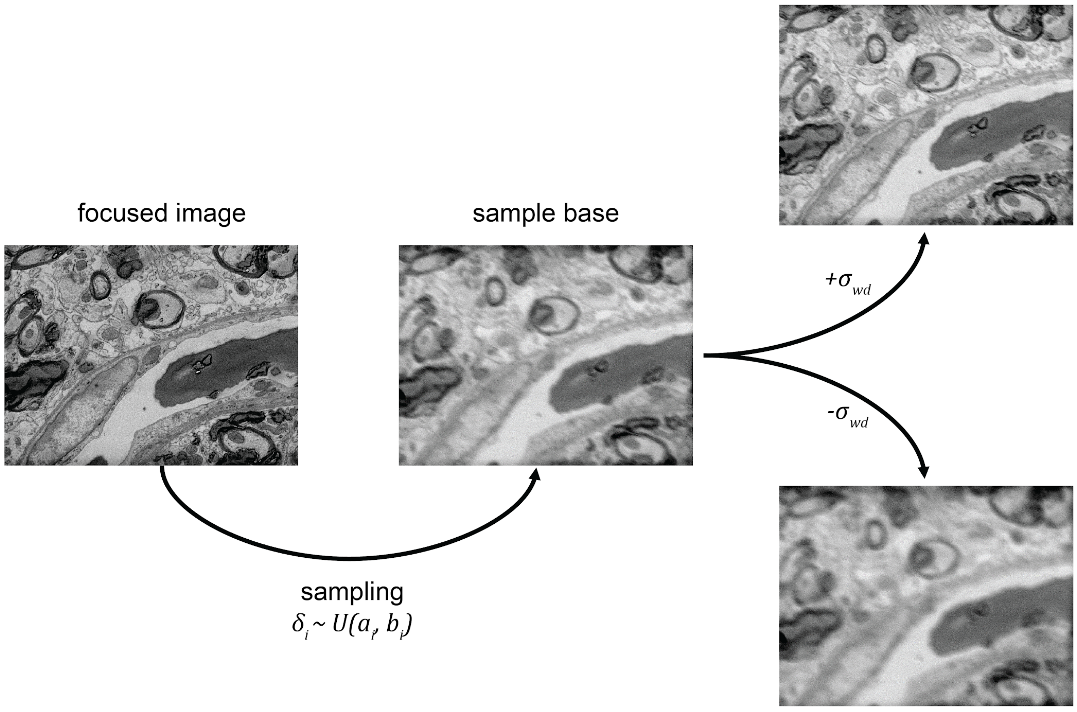


**Supplementary Fig. 1** Training and test data set generation. The focus and stigmator values of the focused image are changed by adding a uniformly and independently sampled offset to generate a set of distorted images and corresponding target values.

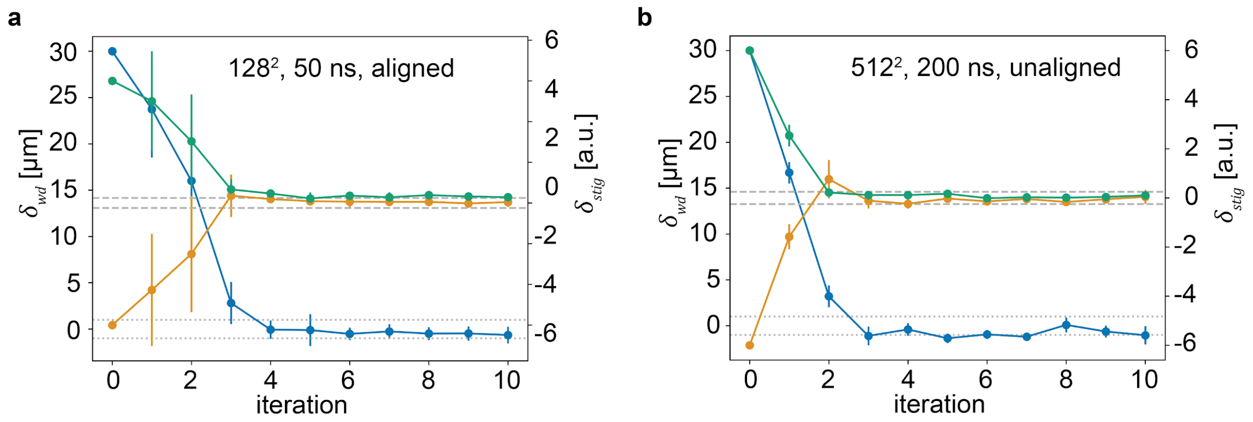


**Supplementary Fig. 2** Convergence of DeepFocus using different input properties. **a** n=20 input patch-pairs (128 × 128) and 50 ns pixel dwell time. **b** n=5 unaligned input patch-pairs (512 × 512) and 200 ns pixel dwell time. Patch locations were drawn independently for each perturbed image. Data in both plots are presented as mean values ± SD. Source data are provided as a Source Data file.

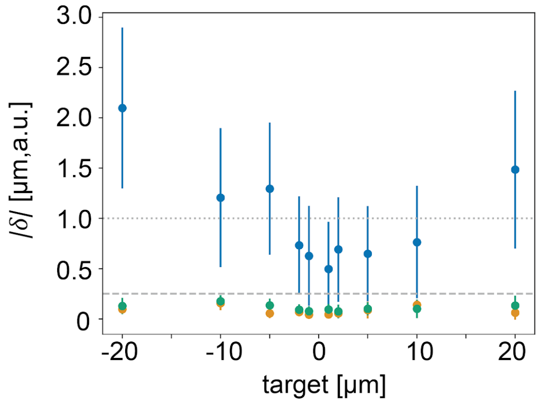


**Supplementary Fig. 3** DeepFocus single-iteration performance as a function of initial defocus. Remaining residual error /*δ*/ between estimate and target from Fig. 2e. Colors as in Fig. 2a. Data are presented as mean values ± SD calculated at n=9 different locations. Source data are provided as a Source Data file.

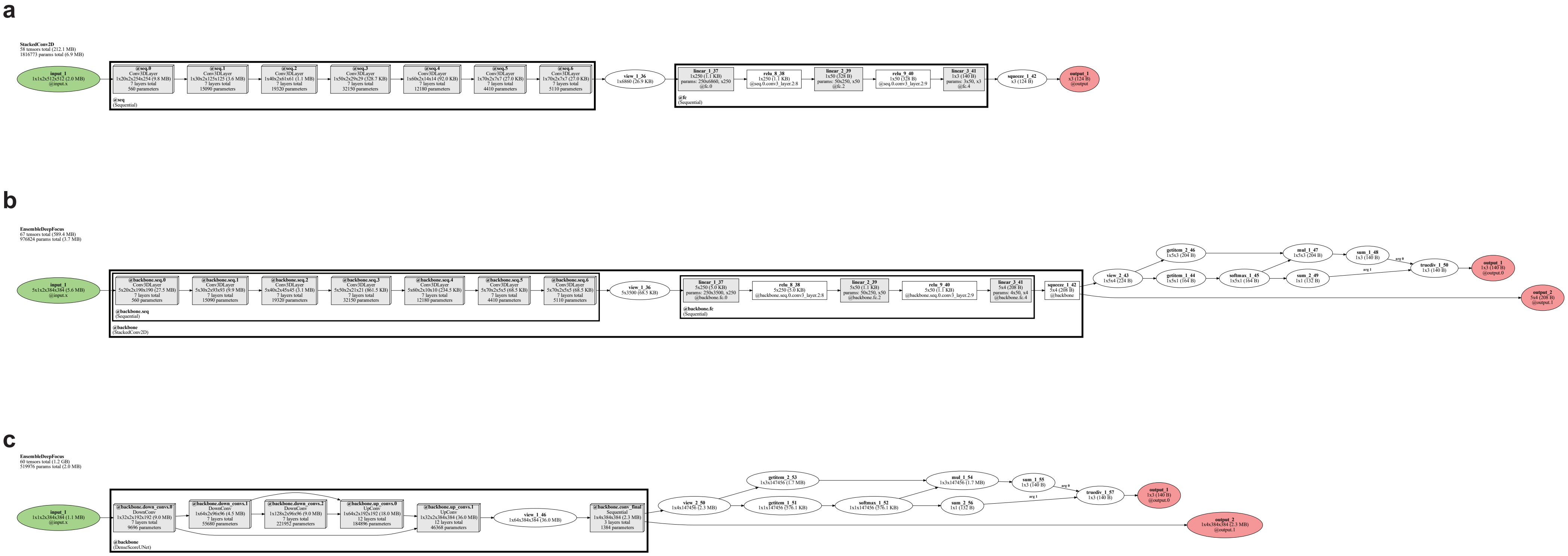


**Supp. Fig. 4** DeepFocus model architectures. **a** Stacked Convolution (image-to-scalar). **b** Stacked Convolution with additional score outputs. **c** U-Net (image-to-image).

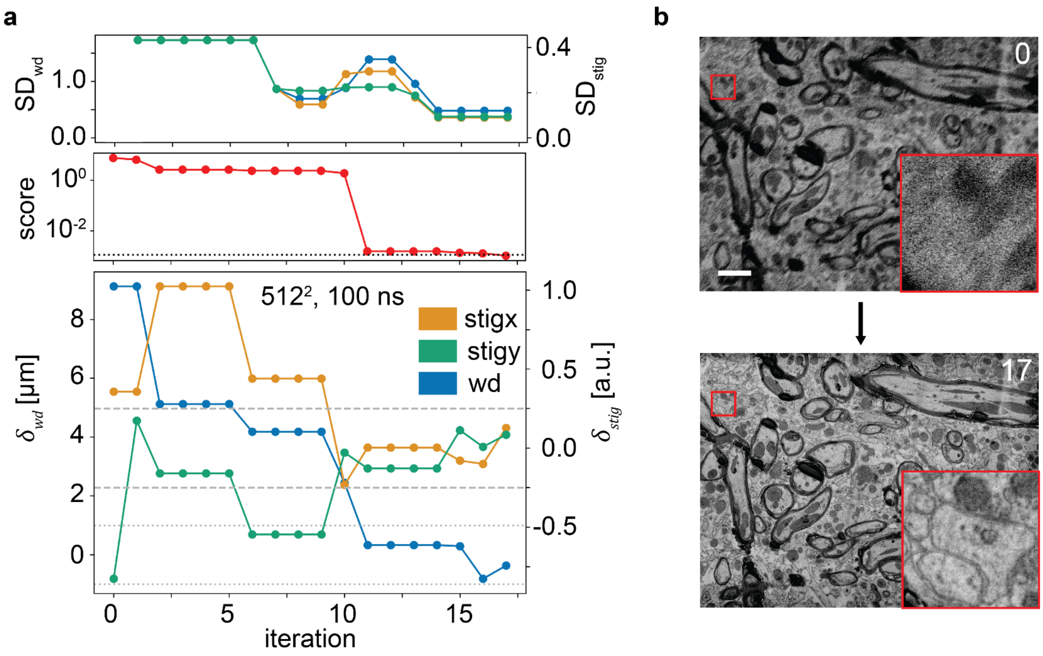


**Supplementary Fig. 5** DeepScore auto-focus evaluation. **a** Convergence of the Nelder-Mead optimization using the DeepScore prediction with n=5 input patch-pairs (512 × 512), 100 ns dwell time, input image size of 2048 × 1568 and a total of 37 score evaluations, i.e. image acquisitions. The focus parameter deviation was calculated as the mean and SD (µm for wd and a.u. for stigmators) from the n=4 simplex vertices for each iteration and parameter (iteration 0 is undefined). **b** Sample images during the Nelder-Mead optimization with the DeepScore objective function from Supp. Fig. 5a at iteration 0 and iteration 17 (introduced aberration: 9.11 µm, 0.35, -0.82). Source data are provided as a Source Data file.

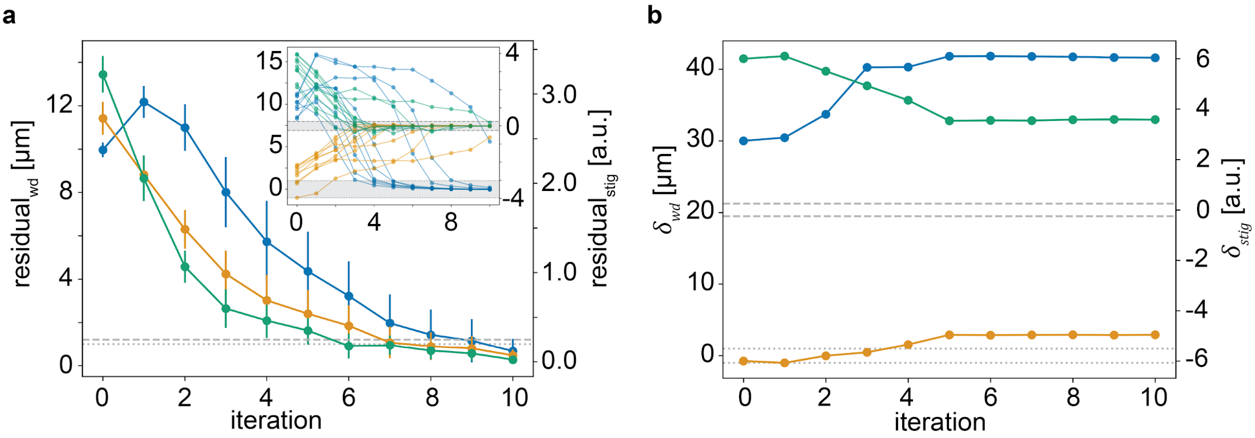


**Supplementary Fig. 6** MAPFoSt convergence with challenging initial defocus parameters. **a** Convergence trajectories with settings as in Fig. 5b, but with 50 ns pixel dwell time. Initial aberrations were sampled as in Fig. 5; baseline focusing was done with MAPFoSt. Data are presented as mean values ± ⅓SD from the n=9 individual trajectories. **b** Single trajectory with initial aberration (30 µm, -6, 6) and 200 ns dwell time. Data are presented as mean values. Source data are provided as a Source Data file.

# Supplementary Tables

**Supplementary Table 1** Single iteration and single input patch-pair performance (mean ± SD) of the stacked convolution model architecture on the neural tissue test data (Methods) using different input patch sizes (squares with edge length 128, 256, 384 or 512).

| Edge length | $MAE_{wd}$ [µm] | $MAE_{stigx}$ [a.u.] | $MAE_{stigy}$ [a.u.] | #Parameters |
|---|---|---|---|---|
| 128 | 5.84 ± 6.11 | 1.04 ± 1.04 | 0.93 ± 0.77 | 108,123 |
| 256 | 3.22 ± 4.27 | 0.68 ± 0.78 | 0.53 ± 0.56 | 416,773 |
| 384 | **2.28 ± 1.74** | **0.43 ± 0.32** | 0.51 ± 0.54 | 976,773 |
| 512 | 2.59 ± 2.39 | 0.45 ± 0.39 | **0.43 ± 0.30** | 1,816,773 |

**Supplementary Table 2** Single iteration and single input patch-pair performance (mean ± SD) of different model architectures on the training dataset (Methods). The Stacked Conv. architecture used an input with an edge length of 512.

| Architecture | $MAE_{wd}$ [µm] | $MAE_{stigx}$ [a.u.] | $MAE_{stigy}$ [a.u.] |
|---|---|---|---|
| Stacked Conv. | 0.63 ± 0.62 | 0.19 ± 0.20 | 0.18 ± 0.18 |
| ResNet-50 | 0.24 ± 0.21 | 0.05 ± 0.06 | 0.05 ± 0.06 |
| EfficientNet-B0 | **0.13 ± 0.13** | **0.05 ± 0.05** | **0.05 ± 0.05** |

**Supplementary Table 3** Single iteration and single input patch-pair performance (mean ± SD) of different model architectures on the validation dataset (Methods). The Stacked Conv. architecture used an input with an edge length of 512.

| Architecture | $MAE_{wd}$ [µm] | $MAE_{stigx}$ [a.u.] | $MAE_{stigy}$ [a.u.] |
|---|---|---|---|
| Stacked Conv. | 1.26 ± 1.43 | 0.30 ± 0.40 | 0.21 ± 0.22 |
| ResNet-50 | 1.13 ± 2.26 | 0.26 ± 0.50 | 0.17 ± 0.21 |
| EfficientNet-B0 | **0.67 ± 0.76** | **0.19 ± 0.23** | **0.16 ± 0.16** |

**Supplementary Table 4** The same as Table 1, but with 10x consensus (average of 10 patch-pair results). The Stacked Conv. architecture used an input with an edge length of 512.

| Architecture | $MAE_{wd}$ [µm] | $MAE_{stigx}$ [a.u.] | $MAE_{stigy}$ [a.u.] |
|---|---|---|---|
| Stacked Conv. | 1.70 ± 1.40 | 0.40 ± 0.26 | 0.38 ± 0.31 |
| ResNet-50 | 3.07 ± 4.61 | 0.50 ± 0.62 | 0.43 ± 0.43 |
| EfficientNet-B0 | **1.26 ± 1.24** | **0.27 ± 0.22** | **0.33 ± 0.29** |

**Supplementary Table 5** Single iteration and single input patch-pair performance (mean ± SD) of different model architectures on tin on carbon test data (Methods). The Stacked Conv. architecture used an input with an edge length of 512.

| Architecture | $MAE_{wd}$ [μm] | $MAE_{stigx}$ [a.u.] | $MAE_{stigy}$ [a.u.] |
|---|---|---|---|
| Stacked Conv. | 3.07 ± 3.28 | 0.57 ± 0.49 | 0.43 ± 0.34 |
| ResNet-50 | 4.02 ± 2.69 | 0.88 ± 0.58 | 0.81 ± 0.65 |
| EfficientNet-B0 | **1.91 ± 1.09** | **0.38 ± 0.29** | **0.34 ± 0.24** |

**Supplementary Table 6** Single iteration and single input patch-pair performance (mean ± SD) of the EfficientNet model architecture with and without pre-training on the neural tissue test data.

| Architecture | $MAE_{wd}$ [μm] | $MAE_{stigx}$ [a.u.] | $MAE_{stigy}$ [a.u.] |
|---|---|---|---|
| EfficientNet-B0 | **1.45 ± 1.43** | **0.29 ± 0.21** | **0.37 ± 0.33** |
| EfficientNet-B0 (w/o pre-training) | 2.24 ± 2.29 | 0.34 ± 0.33 | 0.54 ± 0.59 |

**Supplementary Table 7** Residual error (mean absolute difference from the baseline, see Methods) of nine convergence trajectories of DeepFocus and MAPFoSt (data as in Fig. 5).

| | DeepFocus | | | MAPFoSt | | |
|---|---|---|---|---|---|---|
| Iteration | $MAE_{wd}$ [μm] | $MAE_{stigx}$ [a.u.] | $MAE_{stigy}$ [a.u.] | $MAE_{wd}$ [μm] | $MAE_{stigx}$ [a.u.] | $MAE_{stigy}$ [a.u.] |
| 0 | 10.11 ± 1.03 | 2.72 ± 0.55 | 3.23 ± 0.61 | 10.27 ± 1.01 | 2.79 ± 0.54 | 3.23 ± 0.65 |
| 1 | 3.22 ± 1.42 | 1.04 ± 0.56 | 0.22 ± 0.12 | 9.25 ± 2.36 | 2.39 ± 0.53 | 1.95 ± 0.76 |
| 2 | 0.34 ± 0.31 | 0.17 ± 0.10 | 0.14 ± 0.07 | 7.68 ± 2.85 | 1.73 ± 0.60 | 0.85 ± 0.57 |
| 3 | 0.44 ± 0.34 | 0.10 ± 0.05 | 0.07 ± 0.06 | 4.51 ± 2.75 | 0.83 ± 0.69 | 0.28 ± 0.18 |
| 4 | 0.22 ± 0.13 | 0.06 ± 0.04 | 0.06 ± 0.07 | 2.15 ± 1.87 | 0.28 ± 0.42 | 0.22 ± 0.15 |
| 5 | 0.25 ± 0.18 | 0.07 ± 0.05 | 0.08 ± 0.07 | 0.93 ± 0.77 | 0.08 ± 0.07 | 0.07 ± 0.06 |
| 6 | 0.25 ± 0.19 | 0.09 ± 0.05 | 0.08 ± 0.06 | 0.50 ± 0.21 | 0.05 ± 0.05 | 0.07 ± 0.06 |
| 7 | 0.20 ± 0.15 | 0.07 ± 0.04 | 0.07 ± 0.07 | 0.35 ± 0.18 | 0.05 ± 0.05 | 0.05 ± 0.03 |
| 8 | 0.23 ± 0.17 | 0.05 ± 0.05 | 0.09 ± 0.07 | 0.26 ± 0.19 | 0.05 ± 0.05 | 0.05 ± 0.03 |
| 9 | 0.19 ± 0.12 | 0.05 ± 0.02 | 0.10 ± 0.10 | 0.23 ± 0.17 | 0.05 ± 0.05 | 0.05 ± 0.03 |
| 10 | 0.29 ± 0.21 | 0.07 ± 0.06 | 0.11 ± 0.08 | 0.22 ± 0.14 | 0.05 ± 0.05 | 0.05 ± 0.03 |

**Supplementary Table 8** Mean and standard deviation of mean squared error (MSE; 0.0 is best) and structural similarity index (SSIM; 1.0 is best) of nine convergence trajectories of DeepFocus (Fig. 5a).

| iteration | MSE | SSIM |
|---|---|---|
| 0 | 3875 ± 344 | 0.033 ± 0.002 |
| 1 | 3234 ± 149 | 0.052 ± 0.012 |
| 2 | 3240 ± 256 | 0.060 ± 0.023 |
| 3 | 3162 ± 285 | 0.067 ± 0.025 |
| 4 | 3093 ± 264 | 0.069 ± 0.023 |
| 5 | 3019 ± 240 | 0.073 ± 0.021 |
| 6 | 2949 ± 242 | 0.078 ± 0.021 |
| 7 | 2900 ± 241 | 0.082 ± 0.019 |
| 8 | 2848 ± 223 | 0.086 ± 0.018 |
| 9 | 2840 ± 217 | 0.082 ± 0.018 |
| 10 | 2788 ± 185 | 0.086 ± 0.014 |
